# Myeloid malignancies‐related somatic mutations in aging individuals

**DOI:** 10.1002/mgg3.683

**Published:** 2019-04-21

**Authors:** Diego F. Coutinho, Ilana R. Zalcberg, Bárbara C. R. Monte‐Mór

**Affiliations:** ^1^ Laboratório de Biologia Molecular, Centro de Transplante de Medula Óssea Instituto Nacional de Câncer Rio de Janeiro Brazil; ^2^ Perlmutter Cancer Center New York University Langone Health New York New York

## Abstract

We search for the presence of somatic mutations in 12 genes related to MDS, MPN, and AML in a Brazilian cohort composed of 609 elderly individuals from a census‐based sample.

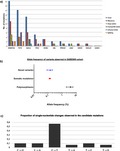

Dear Editor,

Brazilians are a highly heterogeneous population characterized by extensive miscegenation of Europeans, Africans, and Amerindians (Durso et al., 2014) and their genetic background is still underrepresented in the population databases. A recent study used whole exome sequencing (WES) to assess genetic variants of the Brazilian population, focusing on a subset of 609 elderly individuals aged 60 years or more (SABE609 cohort) (Naslavsky et al., 2017). The authors described ancestry markers and novel genetic variants in this group and reported mutations occurring in 56 genes deemed clinically relevant by the American College of Medical Genetics and Genomics. The genomic data obtained from SABE609 cohort were deposited in the Online Archive of Brazilian Mutations (ABraOM) open database.

In addition to germline variants, DNA specimens obtained from aged subjects may also harbor somatic mutations accumulated during their lifetime. In the SABE609 cohort, WES data were obtained from peripheral blood DNA, therefore we hypothesized that the ABraOM database may include somatic mutations related to the aging hematopoiesis.

Aging is associated with higher susceptibility to infections, anemia, and increased risk for hematological neoplasms. It was recently shown that healthy elderly individuals harbor cancer‐related somatic mutations in the blood. Indeed, two independent cohorts were evaluated by WES, one focusing on genes commonly mutated in hematologic cancers (Jaiswal et al., 2014), the other focusing on variants with low variant allelic frequency (VAF) (Genovese et al., 2014). Both studies showed a strong correlation between age and frequency of hematological neoplasm‐associated mutations. Other groups confirmed and extended these results, showing that 10% of individuals >65 years old present those somatic mutations in peripheral blood (McKerrell et al., 2015; Xie et al., 2014; Zink et al., 2017), a condition termed clonal hematopoiesis of indeterminate potential (Steensma et al., 2015). Importantly, these variants were associated with increased risk of hematological malignancies and could represent premalignant events detectable in the blood of healthy individuals (Genovese et al., 2014; Jaiswal et al., 2014).

We herein investigate the presence of potential hematological neoplasm‐associated somatic mutations in peripheral blood of aging Brazilians, using publicly available genomic data deposited in the ABraOM database. For this purpose, we selected 12 genes, recurrently mutated in myeloid malignancies and frequently mutated in four distinct elderly cohorts (Table S1; Figure 1). Searching in the ABraOM databank, a total of 1,633 variants were found in this 12‐gene subset. Variants located within introns, ncRNA and UTR regions were excluded. The remaining 310 variants (19%) were retained for analysis. These variants were classified either as synonymous SNV (*n* = 125), nonsynonymous SNV (missense, *n* = 146), stopgain (nonsense, *n* = 15), insertions and deletions (Indels, *n* = 19), or splicing related (*n* = 5). After excluding synonymous variants, we accessed COSMIC, 1,000 Genomes, and dbSNP databases to classify the other remaining (*n* = 185) into four categories: polymorphisms (41%), candidate somatic mutations (35%), variants of unknown effect (11%), and novel variants (14%).

**Figure 1:**
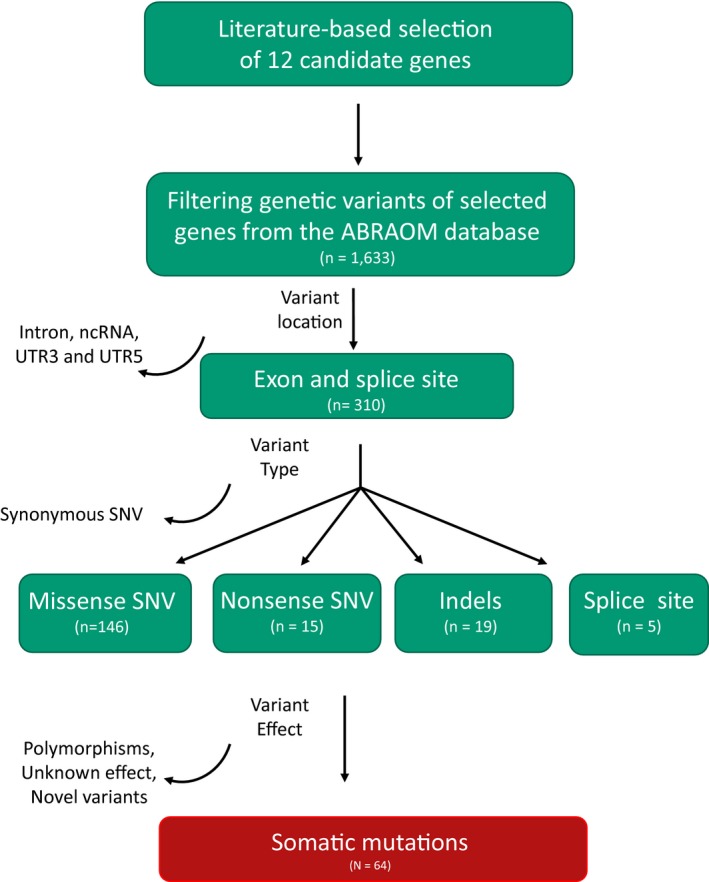
Pipeline used for filtering known myeloid neoplasia‐related mutations from the ABraOM database

We identified 64 variants previously described as somatic mutations affecting 10 out of the 12 analyzed genes (Table S2). Missense mutations were most commonly found in *DNMT3A*, *ASXL1*, *TP53,* and *SF3B1*, while frameshift InDels in *TET2* gene. In accordance with previous reports by other groups (Genovese et al., 2014; Jaiswal et al., 2014; McKerrell et al., 2015), the most frequently mutated genes in aging Brazilians were *DNMT3A*, *TET2,* and *ASXL1,* all three involved in epigenetic regulation (Figure 2a). *DNMT3A* mutations are recurrent in acute myeloid leukemia (AML) and commonly co‐occur with *NPM1* and *FLT3‐ITD* mutations (Grimwade, Ivey, & Huntly, 2016). In the leukemogenesis model, mutations in “landscaping genes” epigenetic regulators such as *DNMT3A*, are early events that happen in a hematopoietic stem cell and drive the expansion of a premalignant clone. Upon subsequent acquisition of mutations in “proliferative genes” such as *FTL3‐TKD,* leukemia develops (Corces‐Zimmerman, Hong, Weissman, Medeiros, & Majeti, 2014; Grimwade et al., 2016). Considering that somatic mutations in the blood of healthy elderly individuals are frequent and increase the risk of developing myeloid neoplasia, it is necessary to distinguish individuals at higher risk. It has been recently shown that, in contrast to the most frequently mutated genes *DNMT3A* and *TET2*, mutations in *TP53* and *U2AF1* confer a high risk of developing AML (Abelson et al., 2018). We found five mutations in *TP53* the ABraOM database, while no mutations in *U2AF1* were observed*.*

**Figure 2:**
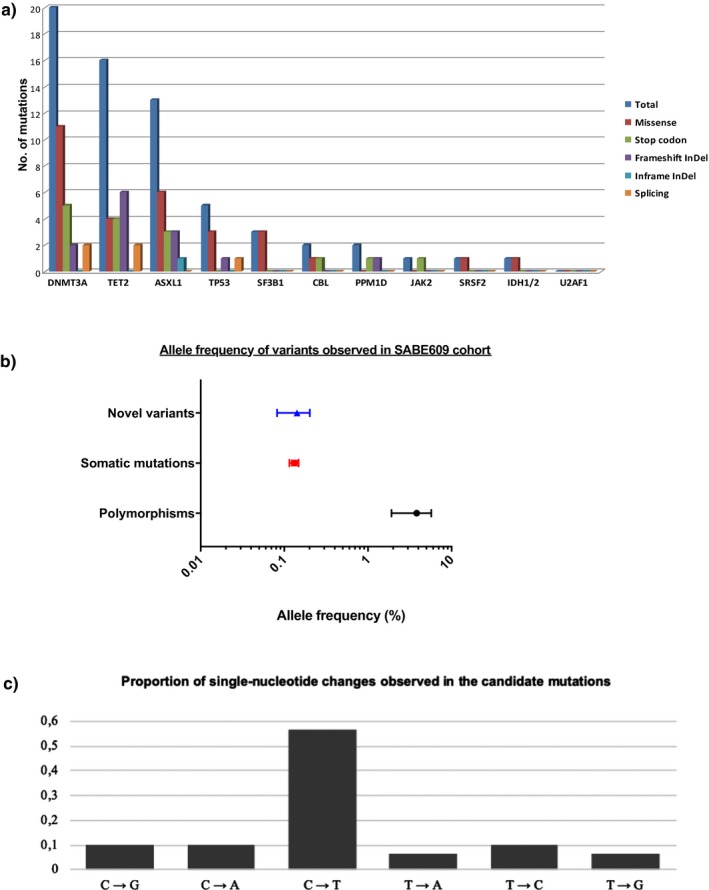
Myeloid neoplasia‐related mutations observed in the ABraOM database. (a) Number and type of mutation by gene. (b) Forest plot of the frequencies in the cohort SABE609 of polymorphisms, mutations, and novel variants. Data were represented by median and standard error of the median (*SEM*) to each variant group. (c) Type of single‐nucleotide changes quantified in the 12‐gene set studied in the ABraOM database

The ABraOM database provides the frequency of each variant in the cohort SABE609. For variants classified as candidate somatic mutation and polymorphism, the median frequencies observed in the Brazilian population were 0.13% (0.08%–0.74%) and 3.5% (0.08%–100%), respectively (Figure 2b). These findings are in accordance with the epidemiological criteria for classification of a genetic variant into polymorphism (≥1%) or mutations (<1%). Novel variants observed in the same cohort showed a median frequency of 0.14% (0.08%–1.56%), suggesting that they may represent putative novel mutations.

On the other hand, individual genomic data of subjects were not available in the ABraOM database. Therefore, number of variants per subject, VAF, mutation co‐occurrence, and the ratio of individuals affected by somatic mutations could not be evaluated in the SABE609 cohort. This information could provide further insight on the clinical significance of these variants, since it has been shown that higher number of mutations per sample and higher VAF are associated with increased risk of developing myeloid neoplasm (Steensma et al., 2015). It has been shown that 5.6% of individuals aged 60–69 years, and 18.4% of individuals over 90 years of age carried somatic mutations (Jaiswal et al., 2014).

Finally, we evaluated the nucleotide substitutions and found that the most frequent change observed was cytosine‐to‐thymine transition (Figure 2c). This substitution is also observed in other elderly cohorts and is prominent in mutational signatures associated with the genetic background of cancers related to aging (Alexandrov et al., 2013). In conclusion, aging Brazilian subjects nonselected for blood cancer might harbor clonal somatic mutations in peripheral blood. Most of genes affected by these candidate somatic variants are involved in epigenetic regulation and may work as founder events in leukemogenesis that are detectable before the disease develops. Since ABraOM study design includes clinical follow‐up and serial blood assessment, future studies should correlate clinical and somatic genomic data.

## FUNDING INFORMATION

DFC was supported by FAPERJ under grant/fellowship no. E‐26/ 202.017/2015.

## CONFLICT OF INTEREST

The authors have declared that no competitive interests exist.
